# Tocilizumab Controls Paraneoplastic Inflammatory Syndrome but Does Not Suppress Tumor Growth of Angiomatoid Fibrous Histiocytoma

**DOI:** 10.1155/2021/5532258

**Published:** 2021-06-16

**Authors:** Hideaki Sabe, Akitomo Inoue, Shigenori Nagata, Yoshinori Imura, Toru Wakamatsu, Satoshi Takenaka, Hironari Tamiya

**Affiliations:** ^1^Department of Orthopedic Surgery, Osaka International Cancer Institute, Osaka 541-8567, Japan; ^2^Department of Diagnostic Pathology and Cytology, Osaka International Cancer Institute, Osaka 541-8567, Japan; ^3^Department of Rehabilitation, Osaka International Cancer Institute, Osaka 541-8567, Japan

## Abstract

Angiomatoid fibrous histiocytoma (AFH) is a rare soft tissue tumor that rarely metastasizes but lacks effective systemic therapy once it propagates. In some reports, high interleukin-6 (IL-6) production promotes tumor growth by autocrine stimulation and tocilizumab, an IL-6 receptor antagonist, can control AFH growth. Here, we present a case report on a patient with local recurrence and distant lymph node metastasis of AFH treated with tocilizumab. As a result, the inhibition of the IL-6 signaling pathway controlled paraneoplastic inflammatory syndrome (PIS); however, the local recurrent tumor progressed. This case implied that IL-6 is not necessarily the cause of tumor growth in AFH. Therefore, physicians should bear in mind that watchful observation is needed whether tocilizumab can control tumor progression despite the amelioration of PIS associated with the attenuated effect of IL-6 on AFH.

## 1. Introduction

Angiomatoid fibrous histiocytoma (AFH) was initially described as angiomatoid malignant fibrous histiocytoma by Enzinger in the late 1970s [1]. AFH often presents in the subcutaneous region in the children and young adults' extremities, with an approximate mean age of 30 years (range: 2 months–71 years) [2]. The World Health Organization's (WHO's) classification categorized AFH as “intermediate tumors of uncertain differentiation [3].” Generally, AFH is nonfatal. However, approximately 15% of patients with AFH develop local recurrence [4]. Therefore, it is often radically resected with a wide margin [5]. Sometimes, unplanned surgery is performed in subcutaneous tumors, including AFH, which may cause local recurrence or distant metastasis [6, 7]. Few studies have reported on the effective treatment for metastatic or unresectable AFH. The occurrence of metastatic and aggressive lesions is usually associated with a fetal outcome. Therefore, a multidisciplinary approach, including surgery, chemotherapy, radiotherapy, and other medical agents, is warranted to control tumor progression in advanced AFH.

Recently, the importance of interleukin-6 (IL-6) in AFH has been highlighted. Some AFH tumors produce IL-6 continuously [8, 9], promoting tumor growth by autocrine stimulation. Tocilizumab, an IL-6 receptor antagonist, which blocks IL-6 binding to the IL-6 receptor, can control tumor progression [10, 11]. Here, we present a recurrent AFH case with nodal metastasis, which we attempted to control using tocilizumab but failed to inhibit tumor progression.

## 2. Case Presentation

**Figure 1:**
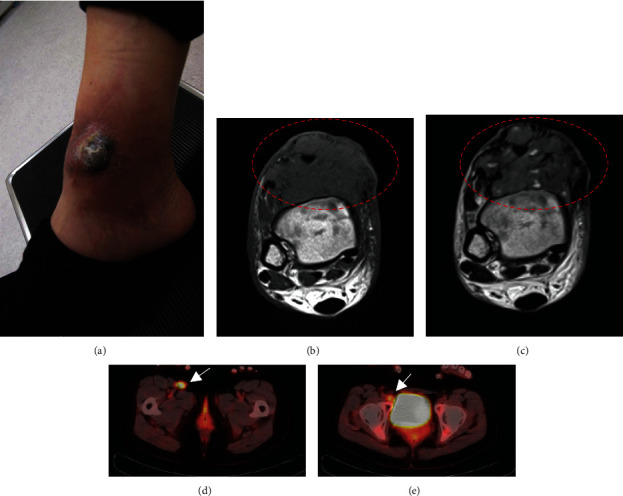
Local recurrence and lymph node metastasis at the initial visit. (a) Visual appearance of the ankle region during the patient's first visit to our hospital. (b) Axial image of the ankle region acquired using T1-weighted MRI. (c) Axial image of the ankle region acquired using T2-weighted MRI. (d) Axial image focused on the inguinal iliac lymph node acquired using positron emission tomography (PET). (e) Axial image focused on the external iliac lymph node acquired using PET.

**Figure 2:**
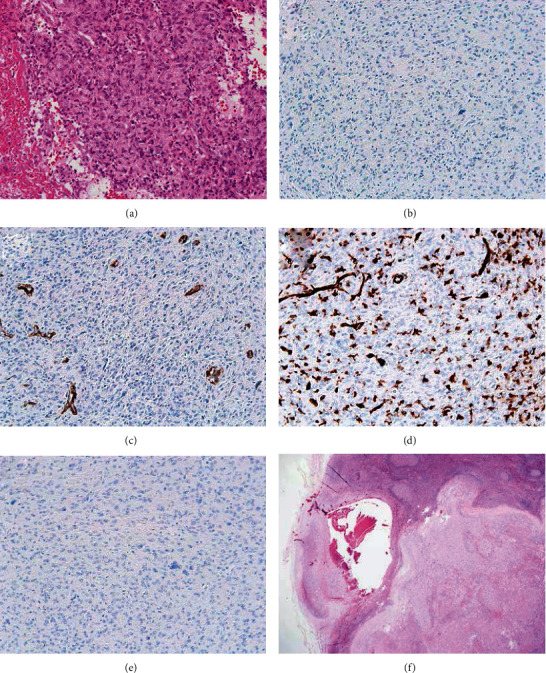
Photomicrographs of the angiomatoid fibrous histiocytoma. (a) Local recurrence featuring solid nodules of epithelioid to spindle cells arranged in a syncytial pattern with peripheral blood cells (hematoxylin and eosin (HE) stain, ×200). Tumor cells negative for (b) S-100 protein, (c) smooth muscle actin, (d) CD31, and (e) STAT6 (immunohistochemical stains, ×200). (f) Excisional biopsy revealing inguinal node metastasis of the tumor (HE, ×20).

**Figure 3:**
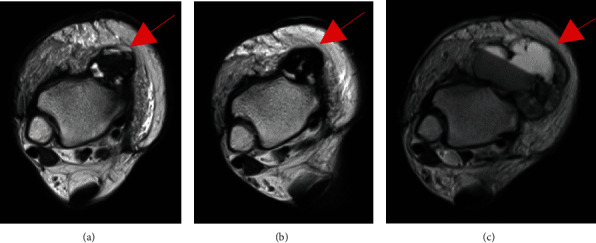
Tumor progression of the local recurrent tumor during tocilizumab treatment. (a) Axial image of the recurrence tumor in the ankle region acquired using T2-weighted MRI at the time of recurrence. (b) Axial image of the recurrence tumor in the ankle region acquired using T2-weighted MRI 2 months after starting on tocilizumab treatment. (c) Axial image of the recurrence tumor in the ankle region acquired using T2-weighted MRI 4 months after the first evaluation. The tumor is indicated by the arrows in red.

A 29-year-old female noticed a small subcutaneous nodule in her right ankle region and underwent tumor resection in 2015. One year later, she recognized a tumor recurrence at the same region. She went to another hospital to undergo tumor resection, with pathological diagnosis of pigmented villonodular synovitis. After a while, she was referred to our hospital because of re-recurrence and came with an approximately 5 cm tumor with skin breakdown in the same region (Figure 1(a)). Magnetic resonance imaging (MRI) revealed a tumor in the ankle region's subcutaneous tissue presenting isointensity and high intensity in the inner cystic region on T1- and T2-weighted images, respectively (Figures 1(b) and 1(c)). Moreover, lymph node enlargement was detected in the right inguinal and external iliac regions (Figures 1(d) and 1(e)). In our institute, histological review revealed AFH. A proliferation of uniform spindle-shaped histiocytoid cells with blunt nuclear atypia and scattered mitoses, accompanying pseudoangiomatous spaces and a pericapsular lymphoplasmacytic rim, was seen with immunohistochemical negativity for S-100 protein, smooth muscle actin (SMA), CD31, and STAT6 (Figures 2(a)–2(e)). Moreover, a chimeric transcript EWSR1 exon 7/ATF1 exon 5 was detected using reverse transcription polymerase chain reaction (primers: EWSR1, forward 5′-TCCTACAGCCAAGCTCCAAGTC-3′; ATF1, reverse 5′-GCCTGGACTTGCCAACTGTAAG-3′). On the first visit to our hospital, the patient's blood test showed C-reactive protein (CRP), hemoglobin (Hb), and IL-6 of 9.84 mg/dl, 9.2 g/dl, and 51.5 pg/ml, respectively, indicating PIS with elevated inflammatory response and mild anemia associated with chronic inflammation (Table 1). The treatment plan was determined by conducting an open biopsy of the right inguinal lymph node swelling, resulting in the diagnosis of lymph node metastasis of AFH (Figure 2(f)). Three months after the first visit, the tumor had been growing rapidly with severe PIS deterioration (Table 1). Therefore, we performed an additional extensive resection of the recurrent tumor with flap reconstruction. The inflammatory response quickly regressed to normal levels following surgery. At 3 and 6 months postoperation, the pelvis lymph node metastasis remained in a stable size and the CRP level increased, respectively. Eight months postoperatively, MRI showed a local recurrence (Figure 3(a)). Because the CRP level increased along with tumor growth, prednisolone (30 mg/day) was initiated to reduce the inflammatory response. However, CRP remained positive and the tumor size continues to grow. Hence, we increased the prednisolone dose to 40 mg/day and considered administering tocilizumab.

**Figure 4:**
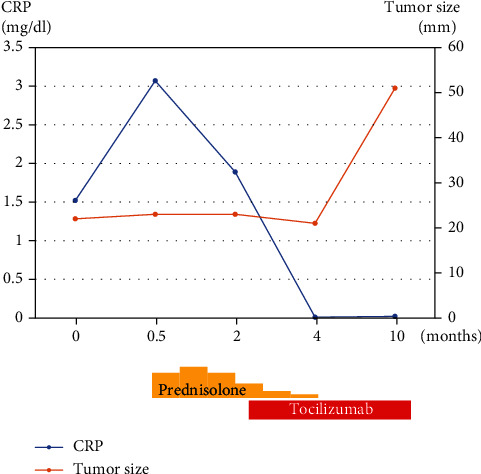
Scheme of the treatment indicating the tumor sizes and CRP levels. The time of tumor recurrence was regarded as time zero.

After knowing the risks and benefits, the patient consented to off-label therapy using tocilizumab, an IL-6 receptor antagonist. She received the subcutaneous injection fortnightly at 162 mg/body. After the treatment started, laboratory parameters normalized within a few weeks, with improved PIS symptoms. Specifically, the CRP level, which was 1.62 mg/dl before the treatment, lowered to less than 0.01 mg/dl in a month and was kept normal. The prescribed prednisolone, which was tapered by 10 mg every 2 weeks in the first 2 months, was eventually discontinued in 3 months. Images showed that both the ankle tumor and external iliac lymph node metastasis retained their size 2 months following the start of tocilizumab treatment (Figure 3(b)). However, the tumor enlarged up to 51 mm 4 months after the first evaluation despite adequate PIS control using tocilizumab. It indicates that tocilizumab had no suppressive effect on AFH growth (Figure 3(c)).  Figure 4 shows the tumor sizes and CRP levels according to the treatment course.

## 3. Discussion

IL-6 was originally identified in the 1970s. Subsequently, it was revealed that IL-6 plays a critical role in inflammatory diseases (e.g., rheumatoid arthritis). An anti-IL-6 receptor antibody, tocilizumab, blocks IL-6 receptor signaling cascade via IL-6 binding to the IL-6 receptor and is commonly used for chronic and acute inflammatory diseases [12]. As previously shown, IL-6 is highly expressed in cancers and a potential promising target but with no approved drugs for cancer therapy so far [13].

AFH is a rare soft tissue tumor, having low-grade malignancy, usually occurring in children and young adults' extremities [5]. Morphologically, it is a multinodular proliferation of bland spindle to ovoid eosinophilic cells, sometimes lining pseudoangiomatous spaces and covered with a thick fibrous pseudocapsule, featuring a pericapsular cuff of prominent lymphoplasmacytic infiltrates. Immunohistochemically, the most relevant finding is the expression of desmin in approximately 40% of cases, suggesting myogenic differentiation [14]. There are three types of characteristic translocation as a causative gene in AFH: t(2;22)(q33;q12) EWSR1-CREB1, t(12;22)(q13;q12) EWSR1-ATF1, and t(12;16)(q13;p11) FUS-ATF1 [8]. Translocations are the initial or early steps in tumor formation, resulting in gene fusions. It frequently leads to the formation of novel, tumor-specific chimeric transcription factors, which can cause gene expression dysregulation [15]. The EWSR1 protein, a member of the TET family, is characterized by a COOH-terminal RNA-binding domain, acting as an adapter between transcription and RNA processing. The CREB protein is a transcription factor regulating cell proliferation, differentiation, and survival [10]. The EWSR1-CREB1 fusion gene, being the most frequent, is constitutively active to promote CREB1 target gene transcription, including IL-6. The IL-6 promoter region includes the binding sites of nuclear factor-kappa B, CREB, CCAAT/enhancer binding protein, and activator protein 1. Whether the EWSR1-ATF1 transcript detected in this tumor can activate the IL-6 gene similarly is unknown. According to a study, an excessive production of IL-6 is detected in AFH with the EWSR1-ATF1 fusion gene, similar to this case [9]. IL-6 triggers STAT3 activation related to cell growth, antiapoptosis, and inflammatory reactions and acts as an autocrine growth factor in a malignant tumor, such as renal cell or prostate cancer [16, 17]. Similarly, IL-6 may also play pivotal roles in both PIS and AFH tumor growth [10]. Thus, patients with AFH may experience systemic symptoms (e.g., pyrexia, anemia, and malaise) due to tumor cytokine production, similar to this case [18]. Several reasons exist for choosing the aforementioned treatment for this case in our hospital. At the time of referral to our hospital by the previous doctor, the patient had an AFH local recurrence and an external iliac lymph node metastasis following resection surgery. Since the tumor was self-destructing, a local surgical intervention was required. Moreover, the tumor produced IL-6, caused inflammatory syndrome, and may be getting bigger, due to IL-6 autocrine stimulation. Considering the patient's general condition, we determined that causative primary tumor removal was necessary. For the lymph node metastasis, we determined that careful follow-up without resection is necessary. First, we hoped that the size will get smaller because of the normalized IL-6 production, owing to primary lesion resection. Second, radical metastasis treatment is difficult. We feared that lymphadenectomy would cause lymphedema, which could affect the patient's ability to perform daily living activity. Therefore, we decided to perform only additional wide resection of the primary tumor. Postoperatively, the CRP levels and IL-6 improved to normal ranges rapidly. The lymph metastasis remained at a stable size. Then, the patient had a tumor recurrence. However, we believe that the aforementioned treatment route was reasonable since we were able to treat the skin's self-destructive area, prevent lymphedema, and temporally improve PIS symptoms.

AFH's standard treatment is surgical resection [18]. Even so, for patients with distant metastasis or frequent local recurrence, similar to this case, radical whole tumor resection may be difficult. According to a study, chemotherapy (e.g., ifosfamide and doxorubicin) can effectively reduce the tumor size of local recurrence or lymph node metastases postoperatively [19]. Conversely, some studies have reported that chemotherapy is ineffective [5]. Until recently, there is little evidence for the efficacy of other treatment modalities on AFH, except for surgery. However, Villiger et al. have reported that tocilizumab may be effective to control AFH growth [11]. As described above, IL-6 is produced continuously in AFH, which may promote tumor growth by autocrine stimulation [10]. Hence, using tocilizumab to block the effects of IL-6 may control AFH growth [11, 20]. Based on these reports, we obtained the patient's consent to try this off-label therapy after explaining its risks and benefits. Tocilizumab was able to suppress tumor growth temporally. However, this effect did not last—eventually, the tumor progressed.

The role of IL-6 in AFH is unclear. However, this case report suggests that IL-6 is not necessarily critical for the AFH growth and controlling tumor growth with tocilizumab only is difficult. Following tocilizumab administration, the CRP level rapidly became normal and PIS symptoms, such as anemia or malaise, improved. In conclusion, tocilizumab can control PIS caused by inhibiting the effects of IL-6, although it does not always suppress tumor growth. In addition, moderate doses of prednisolone could suppress PIS. Considering the side effects of a moderate-dose steroid, tocilizumab was more suitable in suppressing PIS.

According to other studies, tocilizumab treatment for patients with AFH associated with EWSR1-ATF1 translocations did not suppress tumor progression [9]. A difference in tocilizumab's tumor suppression between EWSR1-ATF1 and EWSR1-CREB1 translocations possibly exists. Further investigation is warranted to clarify the difference in these fusion genes.

## Figures and Tables

**Table 1:** The transition of laboratory parameters (e.g., C-reactive protein (CRP), hemoglobin (Hb), albumin (Alb), and interleukin-6 (IL-6)) and the size of the tumor and lymph node in our hospital during the first visit.

	First visit	Before surgery	After surgery	Recurrence	Before tocilizumab	After tocilizumab
CRP (mg/dl)	9.8	18	0.05	2.21	1.89	0.25
Hb (g/dl)	9.2	7.5	12.1	11.3	11.4	10.4
Alb (mg/dl)	3.5	2.3	4	4.2	4.1	3.9
IL-6 (pg/dl)	51.5	N/A	0.935	N/A	12.4	N/A
Tumor size (mm)	49	55	0	19	23	51
Lymph node size (mm)	19	22	21	27	27	27

Before surgery: the primary tumor; after surgery: tumor recurrence; N/A: not applicable.

## Data Availability

There are no available data.
